# Supplementary material for: Complexome profiling of the Chlamydomonas psb28 mutant reveals TEF5 as an early PSII assembly factor
Source: Plant Cell. 2025 Mar 17;37(6):koaf055. doi: 10.1093/plcell/koaf055 (PMC12164590; doi:10.1093/plcell/koaf055)
Supplement: koaf055_Supplementary_Data [file koaf055_supplementary_data.zip › psb28_interactive.zip!/plots/intensity/qualityPlots/peptides/THIC_Cre05.g240850.t1.2.html]

### Cre05.g240850.t1.2

Trivial name: THIC  
SeqCoverage [median]: 1.60  
Mass: 70.45 kDa  
  
 

| Sequence | MissedCleavage | Mass [Da] | wtIntensity | muIntensity | Score |
| --- | --- | --- | --- | --- | --- |
| AGVTQMALAR | 0 | 1016.54 | 357.70 | 785.63 | 113.62 |
|  |  |  | 357.7 | 785.6 |  |
